# Variance Measures for Symmetric Positive (Semi-) Definite Tensors in Two Dimensions

**DOI:** 10.1007/978-3-030-56215-1_1

**Published:** 2021-02-11

**Authors:** Magnus Herberthson, Evren Özarslan, Carl-Fredrik Westin

**Affiliations:** Department of Mathematics, Linköping University, Linköping, Sweden; Department of Biomedical Engineering, Linköping University, Linköping, Sweden; Department of Radiology Brigham and Women’s Hospital, Harvard Medical School, Boston, MA, USA

## Abstract

Calculating the variance of a family of tensors, each represented by a symmetric positive semi-definite second order tensor/matrix, involves the formation of a fourth order tensor *R*_*abcd*_. To form this tensor, the tensor product of each second order tensor with itself is formed, and these products are then summed, giving the tensor *R*_*abcd*_ the same symmetry properties as the elasticity tensor in continuum mechanics. This tensor has been studied with respect to many properties: representations, invariants, decomposition, the equivalence problem et cetera. In this paper we focus on the two-dimensional case where we give a set of invariants which ensures equivalence of two such fourth order tensors *R*_*abcd*_ and ${\tilde{R}}_{abcd}$. In terms of components, such an equivalence means that components *R*_*ijkl*_ of the first tensor will transform into the components ${\tilde{R}}_{ijkl}$ of the second tensor for some change of the coordinate system.

## 1 Introduction

Positive semi-definite second order tensors arise in several applications. For instance, in image processing, a structure tensor is computed from greyscale images that captures the local orientation of the image intensity variations [10, 17] and is employed to address a broad range of challenges. Diffusion tensor magnetic resonance imaging (DT-MRI) [1, 5] characterizes anisotropic water diffusion by enabling the measurement of the apparent diffusion tensor, which makes it possible to delineate the fibrous structure of the tissue. Recent work has shown that diffusion MR measurements of restricted diffusion obscures the fine details of the pore shape under certain experimental conditions [11], and all remaining features can be encoded accurately by a confinement tensor [19].

All such second order tensors share the same mathematical properties, namely, they are real-valued, symmetric, and positive semi-definite. Moreover, in these disciplines, one encounters a collection of such tensors, e.g., at different locations of the image. Populations of such tensors have also been key to some studies aiming to model the underlying structure of the medium under investigation [8, 12, 18].

Irrespective of the particular application, let *R*_*ab*_ denote such tensors,^1^ and we shall refer to the set of *n* tensors as $\{R_{ab}^{\left(i\right)}{\}}_{i}$. Our desire is to find relevant descriptors or models of such a family. One relevant statistical measure of this family is the (population) variance

$$\frac{1}{n}\sum_{i=1}^{n}(R_{ab}^{\left(i\right)}-{\hat{R}}_{ab})(R_{cd}^{\left(i\right)}-{\hat{R}}_{cd})=\left(\frac{1}{n}\sum_{i=1}^{n}R_{ab}^{\left(i\right)}R_{cd}^{\left(i\right)}\right)-{\hat{R}}_{ab}{\hat{R}}_{cd}\;,$$

where ${\hat{R}}_{ab}=\frac{1}{n}\sum_{i=1}^{n}R_{ab}^{\left(i\right)}$ is the mean. (For another approach, see e.g., [8]). In this paper, we are interested in the first term, i.e., we study the fourth order tensor (skipping the normalization)

(1) $$R_{abcd}=\sum_{i=1}^{n}R_{ab}^{\left(i\right)}R_{cd}^{\left(i\right)},\;\;R_{ab}^{\left(i\right)}\geq 0,$$

where $R_{ab}^{\left(i\right)}\geq 0$ stands for $R_{ab}^{\left(i\right)}$ being positive semi-definite. It is obvious that *R*_*abcd*_ has the symmetries *R*_*abcd*_ = *R*_*bacd*_ = *R*_*abdc*_ and *R*_*abcd*_ = *R*_*cdab*_, i.e., *R*_*abcd*_ has the same symmetries as the elasticity tensor [14] from continuum mechanics. The elasticity tensor is well studied [13], e.g. with respect to classification, decompositions, and invariants. In most cases this is done in three dimensions. The same (w.r.t. symmetries) tensor has also been studied in the context of diffusion MR [2].

In this paper we will focus on the corresponding tensor *R*_*abcd*_ in two dimensions. First, there are direct applications in image processing, and secondly, the problems posed will be more accessible in two dimensions than in three. In particular we study the equivalence problem, namely, we ask the question: given the components *R*_*ijkl*_ and ${\tilde{R}}_{ijkl}$ of two such tensors do they represent the same tensor in different coordinate systems (see Sects. 2.1.2 and 4)?

### 1.1 Outline

Section 2 contains tensorial matters. We will assume some basic knowledge of tensors, although some definitions are given for completeness. The notation(s) used is commented on and in particular the three-dimensional Euclidean vector space *V*_(*ab*)_ is introduced.

In Sect. 2.1.2, we make some general remarks concerning the tensor *R*_*abcd*_ and specify the problem we focus on. Section 2.1 is concluded with some remarks on the Voigt/Kelvin notation and the corresponding visualisation in $R^{3}$.

Section 2.2 gives examples of invariants, especially invariants which are easily accessible from *R*_*abcd*_. Also, more general invariant/canonical decompositions of *R*_*abcd*_ are given.

In Sect. 3, we discuss how the tensor *R*_*abcd*_ can (given a careful choice of basis) be expressed in terms of a 3 × 3 matrix, and how this matrix is affected by a rotation of the coordinate system in the underlying two-dimensional space on which *R*_*abcd*_ is defined.

In Sect. 4 we return to the equivalence problem and give the main result of this work. In Sect. 4.1.1 we provide a geometric condition for equivalence, while in Sect. 4.1.2, we present the equivalence in terms of a 3 × 3 matrix. Both these characterisations rely on the choice of particular basis elements for the vector spaces employed. In Sect. 4.1.3 the same equivalence conditions are given in a form which does not assume a particular basis.

## 2 Preliminaries

In this section we clarify the notation and some concepts which we need. Section 2.1 deals with the (alternatives of) tensor notation and some representations. The equivalence (and related) problems are also briefly addressed. Section 2.2 accounts for some natural invariants, traces and decompositions of *R*_*abcd*_.

We will assume some familiarity with tensors, but to clarify the view on tensors we recall some facts. We start with a (finite dimensional) vector space *V* with dual V*. A tensor of order (p,q) is then a multi-linear mapping $\underset{q}{\underset{︸}{V\times V\cdots \times V}}\times \underset{p}{\underset{︸}{V^{*}\times \cdots \times V^{*}}}\to R$. Moreover, a (non-degenerate) metric/scalar product g:V×V→R gives an isomorphism from *V* to V* through *v* → *g*(*v*, ·), and it is this isomorphism which is used to ‘raise and lower indices’, see below. Indeed, for a fixed *v* ∈ *V*, *g*(*v*, ·) is a linear mapping V→R, i.e., an element of V*.

### 2.1 Tensor Notation and Representations

There is a plethora of notations for tensors. Here, we follow the well-adopted convention [16] that early lower case Latin letters (*T*^*a*^_*bc*_) refer to the tensor as a geometric object, its type being inferred from the indices and their positions (the abstract index notation). *g_ab_* denotes the metric tensor. When the indices are lower case Latin letters from the middle of the alphabet, *T*^*i*^_*jk*_, they refer to components of *T*^*a*^_*bc*_ in a certain frame. The super-index *i* denotes a contravariant index while the sub-indices *j, k* are covariant. For instance, a typical vector (tensor of type (1, 0)) will be written *v*^*a*^ with components *v*^*i*^, while the metric *g*_*ab*_ (tensor of type (0, 2)) has components *g*_*ij*_. At a number of occasions, it will also be useful to express quantities in terms of components with respect to orthonormal frames, i.e., Cartesian coordinates. This is sometimes referred to as ‘Cartesian tensors’, and the distinction between contra- and covariant indices is obscured. In these situations, it is possible (but not necessary) to write all indices as sub-indices, and sometimes the symbol ≐ is used to indicate that an equation is only valid in Cartesian coordinates. For example *T*_*i*_ ≐ *T_ijk_δ_jk_* instead of *T*^*i*^ = *T*^*i*^_*jk*_*g*^*jk*^ = *T*^*ik*^_*k*_. Often this is clear form the context, but we will sometimes use ≐ to remind the reader that a Cartesian assumption is made. Here, the Einstein summation convention is implied, i.e., repeated indices are to be summed over, so that for instance $T^{i}=T_{jk}^{i}g^{jk}=T_{\;k}^{ik}=\sum_{j=1}^{n}\sum_{k=1}^{n}T_{jk}^{i}g^{jk}=\sum_{k=1}^{n}T_{\;k}^{ik}$ if each index ranges from 1 to *n*. We have also used the metric *g*_*ij*_ and its inverse *g*^*ij*^ to raise and lower indices. For instance, since *g*_*ij*_*v*^*i*^ is an element of V*, we write *g*_*ij*_*v*^*i*^ = *v_j_*.

We also remind of the notation for symmetrisation. For a two-tensor $T_{\left(ab\right)}=\frac{1}{2}(T_{ab}+T_{ba})$, while more generally for a tensor *T*_*a*_1_*a*_2_⋯*a*_*n*__ of order (0, *n*) we have

$$T_{\left(a_{1}a_{2}\cdots a_{n}\right)}=\frac{1}{n!}\sum_{\pi }T_{a_{\pi (1)}a_{\pi (2)}\cdots a_{\pi (n)}}$$

where the sum is taken over all permutations *π* of 1, 2, …, *n*. Naturally, this convention can also be applied to subsets of indices. For instance, $H_{a(bc)}=\frac{1}{2}(H_{abc}+H_{acb})$.

#### 2.1.1 The Vector Space of Symmetric Two-Tensors

In any coordinate frame a symmetric tensor *R*_*ab*_ (i.e., *R*_*ab*_ = *R_ba_*) is represented by a symmetric matrix *R_ij_* (2 × 2 or 3 × 3 depending on the dimension of the underlying space). In the two-dimensional case, with the underlying vector space $V^{a}∼R^{2}$, this means that *R*_*ab*_ lives in a three-dimensional vector space, which we denote by *V*_(*ab*)_. *V*_(*ab*)_ is equipped with a natural scalar product: < *A*_*ab*_, *B*_*ab*_ >= *A*_*ab*_*B*^*ab*^, making it into a three-dimensional Euclidean space. Here *A*_*ab*_*B*^*ab*^ = *A*_*ab*_*B*_*cd*_*g*^*ac*^*g*^bd^, i.e, the contraction of *A*_*ab*_*B*_*cd*_ over the indices *a, c* and *b, d*, and the tensor product *A*_*ab*_*B*_*cd*_ itself is the tensor of order (0, 4) given by (*A*_*ab*_*B*_*cd*_)*v*^*a*^*u*^*b*^*w*^*c*^*m*^*d*^ = (*A*_*ab*_*v*^*a*^*u*^*b*^)(*B*_*cd*_*w*^*c*^*m*^*d*^) together with multi-linearity.

#### 2.1.2 The Tensor *R*_*abcd*_ and the Equivalence Problem

As noted above, *R*_*abcd*_ given by (1) has the symmetries *R*_*abcd*_ = *R*_(*ab*)*cd*_ = *R*_*ab*(*cd*)_ and *R*_*abcd*_ = *R*_*cdab*_, and it is not hard to see that this gives *R*_*abcd*_ six degrees of freedom in two dimensions. (See also Sect. 2.1.3.) It is also interesting to note that *R*_*abcd*_ provides a mapping *V*_(*ab*)_ → *V*_(*ab*)_ through

$$R_{ab}\mapsto R_{abcd}R^{cd},$$

and that this mapping is symmetric (due to the symmetry *R*_*abcd*_ = *R*_*cdab*_). Given *R*_*abcd*_ there are a number of questions one can ask, e.g.,

- Feasibility—given a tensor *R*_*abcd*_ with the correct symmetries, can it be written in the form (1)?
- Canonical decomposition—given *R*_*abcd*_ of the form (1), can you write *R*_*abcd*_ as a canonical sum of the *form* (1), but with a fixed number of terms (cf. eigenvector decomposition of symmetric matrices)?
- Visualisation—since fourth order tensors are a bit involved, how can one visualise them in ordinary space?
- Characterisation/relevant sets of invariants—what invariants are relevant from an application point of view?
- The equivalence problem—in terms of components, how do we know if *R*_*ijkl*_ and ${\tilde{R}}_{ijkl}$ represent the same tensor when they are in different coordinate systems?

We will now focus on the *equivalence problem* in two dimensions. This problem can be formulated as above: given, in terms of components, two tensors (with the symmetries we consider) *R*_*ijkl*_ and ${\tilde{R}}_{ijkl}$, do they represent the same tensor in the sense that there is a coordinate transformation taking the components *R*_*ijkl*_ into the components ${\tilde{R}}_{ijkl}$? In other words, does there exist an (invertible) matrix *P*^*m*^_*i*_ so that

$$R_{ijkl}={\tilde{R}}_{mnop}{P^{m}}_{i}{P^{n}}_{j}{P^{o}}_{k}{P^{p}}_{l}?$$

This problem can also be formulated when *R*_*ijkl*_ and ${\tilde{R}}_{ijkl}$ are expressed in Cartesian frames. Then the coordinate transformation must be a rotation, i.e., given by a rotation matrix *Q*^*i*^ *j* ∈ SO(2). Hence, the problem of (unitary) equivalence is: Given *R*_*ijkl*_ and ${\tilde{R}}_{ijkl}$, both expressed in Cartesian frames, is there a matrix (applying the ‘Cartesian convention’) *Q*_*ij*_ ∈ SO(2) so that

$$R_{ijkl}={\tilde{R}}_{mnop}Q_{mi}Q_{nj}Q_{ok}Q_{pl}?$$

#### 2.1.3 The Voigt/Kelvin Notation

Since (in two dimensions) the space *V*_(*ab*)_ is three-dimensional, one can introduce coordinates, for example $R_{ij}=\left(\begin{array}{cc} x & y\\ y & z \end{array}\right)∼\left(\begin{array}{c} x\\ y\\ z \end{array}\right)$ and use vector algebra on $R^{3}$. This is used in the Voigt notation [15] and the related Kelvin notation [6]. As always, one must be careful to specify with respect to which basis in *V*_(*ab*)_ the coordinates $\left(\begin{array}{c} x\\ y\\ z \end{array}\right)$ are taken. For instance, in the correspondence $R_{ij}=\left(\begin{array}{cc} x & y\\ y & z \end{array}\right)∼\left(\begin{array}{c} x\\ y\\ z \end{array}\right)$, the understood basis for *V*_(*ab*)_ (in the understood/induced coordinate system) is $\left\{\left(\begin{array}{cc} 1 & 0\\ 0 & 0 \end{array}\right),\left(\begin{array}{cc} 0 & 1\\ 1 & 0 \end{array}\right),\left(\begin{array}{cc} 0 & 0\\ 0 & 1 \end{array}\right)\right\}$. These elements are orthogonal (viewed as vectors in *V*_(*ab*)_) to each other, but not (all of them) of unit length.

Since the unit matrix plays a special role, we make the following choice. Starting with an orthonormal basis $\left\{\hat{\xi },\hat{\eta }\right\}$ for *V*, (i.e., $\left\{{\hat{\xi }}^{a},{\hat{\eta }}^{a}\right\}$ for *V*^*a*^) a suitable orthonormal basis for *V*_(*ab*)_ is $\left\{e_{ab}^{\left(1\right)},e_{ab}^{\left(2\right)},e_{ab}^{\left(3\right)}\right\}$ where $e_{ab}^{\left(1\right)}=\frac{1}{\sqrt{2}}(\xi_{a}\xi_{b}-\eta_{a}\eta_{b})$, $e_{ab}^{\left(2\right)}=\frac{1}{\sqrt{2}}(\xi_{a}\eta_{b}+\eta_{a}\xi_{b})$, $e_{ab}^{\left(3\right)}=\frac{1}{\sqrt{2}}(\xi_{a}\xi_{b}+\eta_{a}\eta_{b})$, i.e., in the induced basis we have

(2) $$e_{ij}^{\left(1\right)}=\frac{1}{\sqrt{2}}\left(\begin{array}{cc} 1 & 0\\ 0 & -1 \end{array}\right)∼\hat{x},\;\;e_{ij}^{\left(2\right)}=\frac{1}{\sqrt{2}}\left(\begin{array}{cc} 0 & 1\\ 1 & 0 \end{array}\right)∼\hat{y},\;\;e_{ij}^{\left(3\right)}=\frac{1}{\sqrt{2}}\left(\begin{array}{cc} 1 & 0\\ 0 & 1 \end{array}\right)∼\hat{z}.$$

In this basis, we write an arbitrary element *M*_*ab*_ ∈ *V*_(*ab*)_ as $M_{ij}=\left(\begin{array}{cc} z+x & y\\ y & z-x \end{array}\right)$, which means that *M*_*ab*_ gets the coordinates ${\bar{M}}_{i}=\sqrt{2}\left(\begin{array}{c} x\\ y\\ z \end{array}\right)$. Note that *M*_*ij*_ is positive definite if *z*^2^ − *x*^2^ − *y*^2^ ≥ 0 and *z* ≥ 0. In terms of the coordinates of the Voigt notation, the tensor *R*_*abcd*_ corresponds to a symmetric mapping $R^{3}\to R^{3}$, given by a symmetric 3 × 3 matrix, which also shows that the degrees of freedom for *R*_*abcd*_ is six.

#### 2.1.4 Visualization in $R^{3}$

Through the Voigt notation, any symmetric two-tensor (in two dimensions) can be visualised as a vector in $R^{3}$. Using the basis vector given by (2), we note that $e_{ij}^{\left(1\right)}$ and $e_{ij}^{\left(2\right)}$ correspond to indefinite quadratic forms, while $e_{ij}^{\left(3\right)}$ is positive definite. We also see that $e_{ij}^{\left(1\right)}+e_{ij}^{\left(3\right)}$ and $e_{ij}^{\left(2\right)}+e_{ij}^{\left(3\right)}$ are positive semi-definite.

**Fig. 1:**
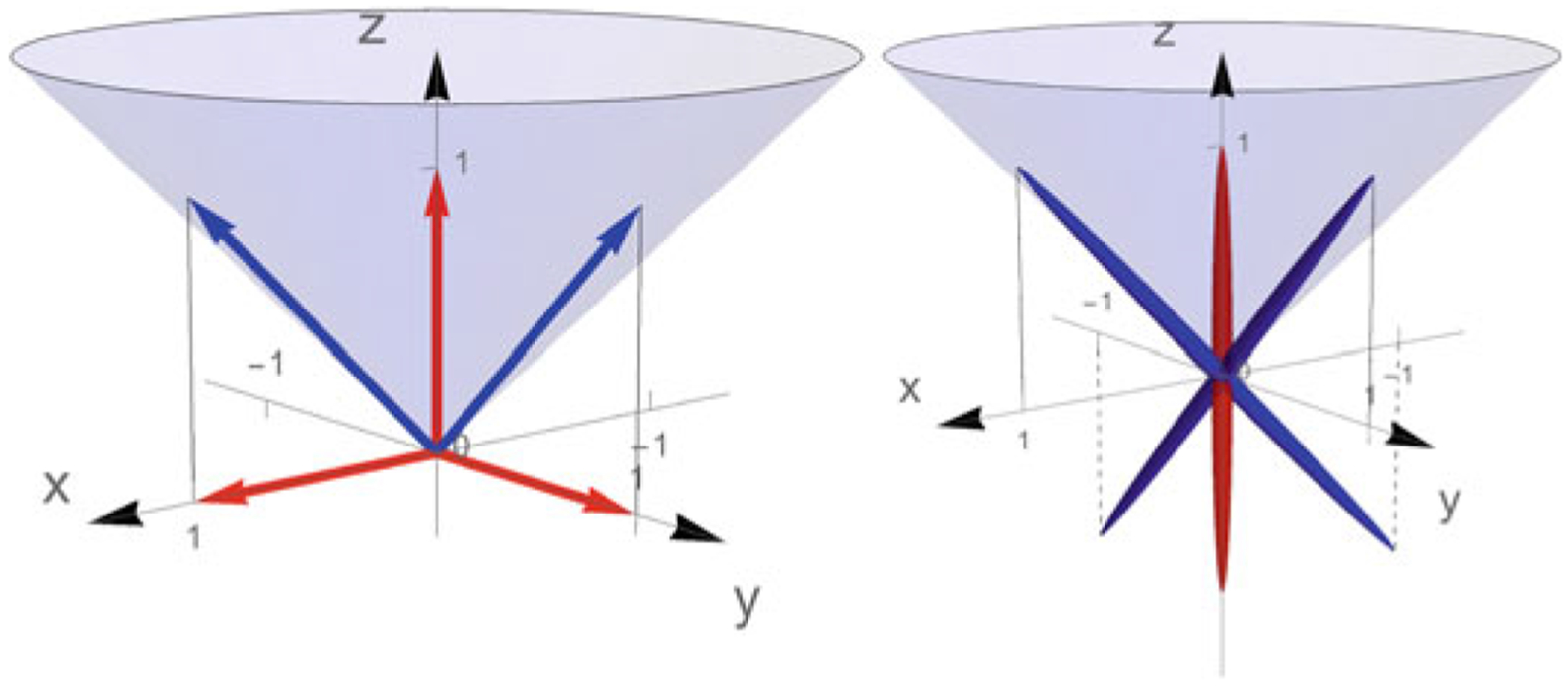
Left: the symmetric matrices $e_{ab}^{\left(1\right)}$, $e_{ab}^{\left(2\right)}$, $e_{ab}^{\left(3\right)}$ (red) and $e_{ab}^{\left(1\right)}+e_{ab}^{\left(3\right)}$, $e_{ab}^{\left(2\right)}+e_{ab}^{\left(3\right)}$ (blue) as vectors in $R^{3}$. The positive semi-definite matrices correspond to vectors which are inside/above the indicated cone (including the boundary). Right: the fourth order tensors $\left(e_{ab}^{\left(1\right)}+e_{ab}^{\left(3\right)})(e_{cd}^{\left(1\right)}+e_{cd}^{\left(3\right)}\right)$ and $\left(e_{ab}^{\left(2\right)}+e_{ab}^{\left(3\right)})(e_{cd}^{\left(2\right)}+e_{cd}^{\left(3\right)}\right)$ depicted in blue, and $e_{ab}^{\left(3\right)}e_{cd}^{\left(3\right)}$ shown in red are viewed as quadratic forms and illustrated as ellipsoids (made a bit ‘fatter’ than they should be for the sake of clarity)

**Fig. 2:**
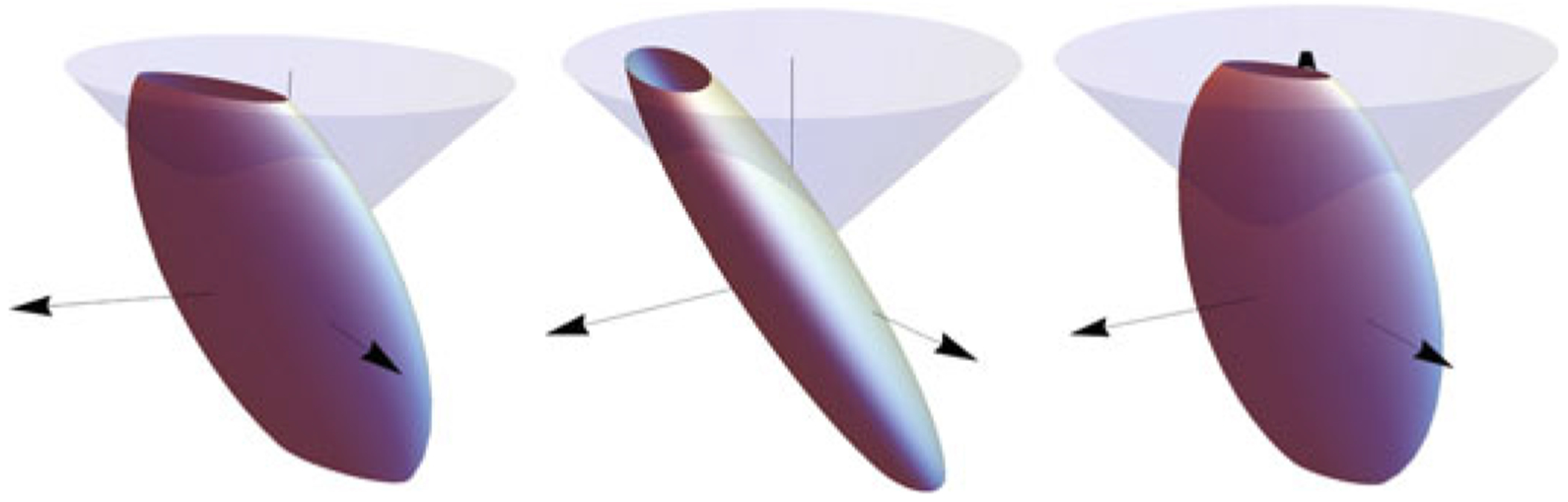
Three identical (truncated) ellipsoids in $R^{3}$ with different orientations. The two leftmost ellipsoids can be carried over to each other through a rotation around the (vertical in the figure) *z*-axis, which implies that they represent the same tensor *R*_*abcd*_ (up to the meaning discussed). The right ellipsoid, despite identical eigenvalues with the two others, represent a different tensor since the rotation which carries this ellipsoid to any of the others is not around the *z*-axis

In Fig. 1 (left) these matrices are illustrated as vectors in $R^{3}$. The set of positive semi-definite matrices corresponds to a cone, cf. [4], indicated in blue. When the symmetric 2 × 2 matrices are viewed as vectors in $R^{3}$, the outer product of such a vector with itself gives a symmetric 3 × 3 matrix. Hence we get a positive semi-definite quadratic form on $R^{3}$, which can be illustrated by an (degenerate) ellipsoid in $R^{3}$. In Fig. 1 (right) $\left(e_{ab}^{\left(1\right)}+e_{ab}^{\left(3\right)})(e_{cd}^{\left(1\right)}+e_{cd}^{\left(3\right)}\right)$, $\left(e_{ab}^{\left(2\right)}+e_{ab}^{\left(3\right)})(e_{cd}^{\left(2\right)}+e_{cd}^{\left(3\right)}\right)$ and $e_{ab}^{\left(3\right)}e_{cd}^{\left(3\right)}$ are visualised in this manner. Note that all these quadratic forms correspond to matrices which are rank one. (Cf. the ellipsoids in Fig. 2.)

### 2.2 Invariants, Traces and Decompositions

By an invariant, we mean a quantity that can be calculated from measurements, and which is independent of the frame/coordinate system with respect to which the measurements are performed, despite the fact that components, e.g., *T*^*i*^_*jk*_ themselves depend on the coordinate system. It is this property that makes invariants important, and typically they are formed via tensor products and contractions, e.g., *T*^*i*^_*jk*_*T*^*k*^_*il*_*g*^*jl*^. Sometimes, the invariants have a direct geometrical meaning. For instance, for a vector *v*^*i*^, the most natural invariant is its squared length *v*^*i*^*v*_*i*_. For a tensor *T*^*i*^_*j*_ of order (1,1) in three dimensions, viewed as a linear mapping $R^{3}\to R^{3}$, the most well known invariants are perhaps the trace *T*^*i*^_*i*_ and the determinant det(*T*^*i*^_*j*_). The modulus of the determinant gives the volume scaling under the mapping given by *T*^*i*^_*j*_, while the trace equals the sum of the eigenvalues. If *T*^*i*^_*j*_ represents a rotation matrix, then its trace is 1 + 2 cos *ϕ*, where *ϕ* is the rotation angle. In general, however, the interpretation of a given invariant may be obscure. (For an account relevant to image processing, see e.g., [9]. A different, but relevant, approach in the field of diffusion MRI is found in [20].)

#### 2.2.1 Natural Traces and Invariants

From (1), and considering the symmetries of *R_abcd_*, two (and only two) natural traces arise. For a tensor of order (1, 1), e.g., *R*_*i*_^*j*^, it is natural to consider this as an ordinary matrix, and consequently use stem letters without any indices at all. To indicate this slight deviation from the standard tensor notation, we denote e.g., *R*_*i*_^*j*^ by $\bar{\bar{R}}$. Using [·] for the trace, so that $[\bar{\bar{R}}]=\text{Tr}(\bar{\bar{R}})=R_{a}^{\;\;a}$, we then have

(3) $$T_{ab}=R_{abc}^{\;\;\;c}=\sum_{i=1}^{n}R_{ab}^{\left(i\right)}R_{c}^{(i{)}^{c}}=\sum_{i=1}^{n}R_{ab}^{\left(i\right)}[{\bar{\bar{R}}}^{\left(i\right)}],$$

and

(4) $$S_{ab}=R_{acb}^{\;\;\;c}=\sum_{i=1}^{n}R_{ac}^{\left(i\right)}R_{b}^{(i{)}^{c}}.$$

Hence, in a Cartesian frame, where the index position is unimportant, we have for the matrices $\bar{\bar{T}}=T_{ij}$, $\bar{\bar{S}}=S_{ij}$

$$\bar{\bar{T}}=\sum_{i=1}^{n}{\bar{\bar{R}}}^{\left(i\right)}[{\bar{\bar{R}}}^{\left(i\right)}],\;\;\bar{\bar{S}}=\sum_{i=1}^{n}{\bar{\bar{R}}}^{\left(i\right)}{\bar{\bar{R}}}^{\left(i\right)}.$$

To proceed there are two double traces (i.e., contracting *R_abcd_* twice):

(5) $$T=T_{a}^{\;a}={R_{a}^{\;\;a}}_{c}^{\;\;c}=\sum_{i=1}^{n}R_{a}^{(i{)}^{a}}R_{c}^{(i{)}^{c}}=\sum_{i=1}^{n}[{\bar{\bar{R}}}^{\left(i\right)}{]}^{2}$$

and

(6) $$S=S_{a}^{\;\;a}=R_{ac}^{\;\;\;ac}=\sum_{i=1}^{n}R_{ac}^{\left(i\right)}R^{(i{)}^{ac}}=\sum_{i=1}^{n}[({\bar{\bar{R}}}^{\left(i\right)}{)}^{2}].$$

In two dimensions, the difference *T*_*ab*_−*S*_*ab*_ is proportional to the metric *g*_*ab*_. Namely,

**Lemma 1** *With T_ab_ and S_ab_ given by* (3) *and* (4), *it holds that (in two dimensions)*

$$T_{ab}-S_{ab}=\sum_{i=1}^{n}\text{det}({\bar{\bar{R}}}^{\left(i\right)})g_{ab}.$$

***Proof*** By linearity, it is enough to prove the statement when *n* = 1, i.e., when the sum has just one term. Raising the second index, and using components, the statement then is $T_{i}^{\;j}-S_{i}^{\;j}=\text{det}({\bar{\bar{R}}}^{\left(1\right)})\delta_{i}^{\;j}$. Putting ${\bar{\bar{R}}}^{\left(1\right)}=A$, we see that *T*_*i*_^*j*^ − *S*_*i*_^*j*^ = *A*[*A*] − *A*^2^ while $\text{det}({\bar{\bar{R}}}^{\left(1\right)})\delta_{i}^{\;j}=\text{det}(A)I$, and by the Cayley-Hamilton theorem in two dimensions, *A*[*A*] − *A*^2^ is indeed det(*A*)*I*. □

From lemma 1, it follows that $T-S=2\sum_{i=1}^{n}\text{det}({\bar{\bar{R}}}^{\left(i\right)})\geq 0$. In fact the following inequalities hold.

**Lemma 2** *With T and S defined as above, it holds that S* ≤ *T* ≤ 2*S*. *If T = S, all tensors* $R_{ab}^{\left(i\right)}$ *have rank 1. If T* = 2*S, all tensors* $R_{ab}^{\left(i\right)}$ *are isotropic, i.e., proportional to the metric g_ab_*.

***Proof*** Again, by linearity it is enough to consider one tensor ${\bar{\bar{R}}}^{\left(1\right)}=A$. In an orthonormal frame which diagonalises *A*, we have $A=\left(\begin{array}{cc} a & 0\\ 0 & c \end{array}\right)$ (with *a* ≥ 0, *c* ≥ 0, *a* + *c* > 0). Hence

$$S=a^{2}+c^{2}\leq a^{2}+c^{2}+2ac=(a+c{)}^{2}=T=2(a^{2}+c^{2})-(a-c{)}^{2}\leq 2S.$$

The first inequality becomes equality when *ac* = 0, i.e., when *A* has rank one. The second inequality becomes equality when *a* = *c*, i.e., when *A* is isotropic. □

**Definition 1** We define the mean rank, *r_m_*, by *r*_*m*_ = *T/S*, with *T* and *S* as above. Hence, in two dimensions, 1 ≤ *r*_*m*_ ≤ 2.

#### 2.2.2 A Canonical Decomposition

It is customary [3, 7] to decompose a tensor with the symmetries of *R*_*abcd*_ into a sum where one term is the completely symmetric part:

$$R_{abcd}=H_{abcd}+W_{abcd},\;\text{where}\;H_{abcd}=R_{\left(abcd\right)},\;W_{abcd}=R_{abcd}-H_{abcd}.$$

It is also customary to split *H*_*abcd*_ into a trace-free part and ‘trace part’. We start by defining *H*_*ab*_ = *H*_*abc*_^*c*^, *H* = *H*_*a*_^*a*^ and then the trace-free part of $H_{ab}\;:{\overset{{}_{\;{}_{\circ }}}{H}}_{ab}=H_{ab}-\frac{1}{2}Hg_{ab}$ so that $H_{ab}={\overset{{}_{\;{}_{\circ }}}{H}}_{ab}+\frac{1}{2}Hg_{ab}$. (These decompositions can be made in any dimension, but the actual coefficients, e.g., $\frac{1}{2}$ above and $\frac{1}{8}$ and $\frac{3}{8}$ et cetera below depend on the underlying dimension.) It is straightforward to check that

$${\overset{\;{}_{{}_{\circ }}}{H}}_{abcd}=H_{abcd}-g_{(ab}H_{cd)}+\frac{1}{8}\;Hg_{(ab}g_{cd)}=H_{abcd}-g_{(ab}{\overset{\;{}_{{}_{\circ }}}{H}}_{cd)}-\frac{3}{8}\;Hg_{(ab}g_{cd)}$$

is also trace-free. Hence we have the decomposition

$$H_{abcd}={\overset{\;{}_{{}_{\circ }}}{H}}_{abcd}+g_{(ab}H_{cd)}-\frac{1}{8}\;H\;g_{(ab}g_{cd)}={\overset{\;{}_{{}_{\circ }}}{H}}_{abcd}+g_{(ab}{\overset{\;{}_{{}_{\circ }}}{H}}_{cd)}+\frac{3}{8}\;H\;g_{(ab}g_{cd)}.$$

Moreover, due to the symmetry of *R*_*abcd*_, we find that

$$H_{abcd}=\frac{1}{3}\;(R_{abcd}+R_{acbd}+R_{adbc})$$

and therefore that

(7) $$W_{abcd}=\frac{1}{3}\;(2R_{abcd}-R_{acbd}-R_{adbc})$$

which implies that $H_{ab}=H_{abc}^{\;\;\;c}=\frac{1}{3}(T_{ab}+2S_{ab})$ and $W_{ab}=W_{abc}^{\;\;\;c}=\frac{2}{3}(T_{ab}-S_{ab})$.

The degres of freedom, which for *R*_*abcd*_ is six, is distributed, where $R_{abcd}∼\{{\overset{{}_{\;{}_{\circ }}}{H}}_{abcd},H_{ab},W_{abcd}\}$, as

$$\underset{\left(6\right)}{R_{abcd}}∼\{\underset{\left(2\right)}{{\overset{\;{}_{{}_{\circ }}}{H}}_{abcd}},\underset{\left(3\right)}{H_{ab}},\underset{\left(1\right)}{W_{abcd}}\}∼\{\underset{\left(2\right)}{{\overset{\;{}_{{}_{\circ }}}{H}}_{abcd}},\underset{\left(2\right)}{{\overset{\;{}_{{}_{\circ }}}{H}}_{ab}},\underset{\left(1\right)}{H},\underset{\left(1\right)}{W_{abcd}}\}.$$

For *H*_*ab*_ (or the pair ${\overset{{}_{\;{}_{\circ }}}{H}}_{ab}$, *H*) this is clear. The total symmetry of ${\overset{{}_{\;{}_{\circ }}}{H}}_{abcd}$ leaves only five components (in a basis), ${\overset{{}_{\;{}_{\circ }}}{H}}_{1111}$, ${\overset{{}_{\;{}_{\circ }}}{H}}_{1112}$, ${\overset{{}_{\;{}_{\circ }}}{H}}_{1122}$, ${\overset{{}_{\;{}_{\circ }}}{H}}_{1222}$, ${\overset{{}_{\;{}_{\circ }}}{H}}_{2222}$. However, the trace-free condition ${\overset{{}_{\;{}_{\circ }}}{H}}_{abcd}\;g^{cd}=0$ imposes three conditions. (In an orthonormal frame, ${\overset{{}_{\;{}_{\circ }}}{H}}_{1122}=-{\overset{{}_{\;{}_{\circ }}}{H}}_{1111}$, ${\overset{{}_{\;{}_{\circ }}}{H}}_{2222}=-{\overset{{}_{\;{}_{\circ }}}{H}}_{1122}$ and ${\overset{{}_{\;{}_{\circ }}}{H}}_{1112}=-{\overset{{}_{\;{}_{\circ }}}{H}}_{1222}$.) That *W_abcd_* has only one degree of freedom follows from the following lemma.

**Lemma 3** *Suppose that W_abcd_ is given by* (7), *and put W_ab_ = W_abcd_g^cd^, W = W_ab_g^ab^. Then (in two dimensions)*

$$W_{abcd}=\frac{W}{4}\;(2g_{ab}g_{cd}-g_{ac}g_{bd}-g_{ad}g_{bc})$$

***Proof*** By linearity, it is enough to consider the case when *R*_*abcd*_ = *A*_*ab*_*A*_*cd*_ for some (symmetric) *A_ab_*. In terms of eigenvectors (to *A^a^_b_*) we can write *A*_*ab*_ = *αx*_*a*_*x*_*b*_ + *βy*_*a*_*y*_*b*_, where *x_a_x*^*a*^ = *y*_*a*_*y*^*a*^ = 1, *x_a_y*^*a*^ = 0. In particular *g_ab_* = *x_a_x_b_* + *y_a_y_b_*. From (7) we then get

(8) $$W_{abcd}=\frac{1}{3}\;(2R_{abcd}-R_{acbd}-R_{adbc})\;\;=\frac{1}{3}\;(2A_{ab}A_{cd}-A_{ac}A_{bd}-A_{ad}A_{bc})\;\;=\frac{1}{3}\;(2(\alpha x_{a}x_{b}+\beta y_{a}y_{b})(\alpha x_{c}x_{d}+\beta y_{c}y_{d})\;\;-(\alpha x_{a}x_{c}+\beta y_{a}y_{c})(\alpha x_{b}x_{d}+\beta y_{b}y_{d})\;\;-(\alpha x_{a}x_{d}+\beta y_{a}y_{d})(\alpha x_{b}x_{c}+\beta y_{b}y_{c})).$$

Expanding the parentheses, the components *x*_*a*_*x*_*b*_*x*_*c*_*x*_*d*_ and *y*_*a*_*y*_*b*_*y*_*c*_*y*_*d*_ vanish, leaving

(9) $$\frac{\alpha \beta }{3}(2x_{a}x_{b}y_{c}y_{d}+2y_{a}y_{b}x_{c}x_{d}-x_{a}x_{c}y_{b}y_{d}\;\;-y_{a}y_{c}x_{b}x_{d}-x_{a}x_{d}y_{b}y_{c}-y_{a}y_{d}x_{b}x_{c})\;=\frac{\alpha \beta }{3}\;(2g_{ab}g_{cd}-g_{ac}g_{bd}-g_{ad}g_{bc})\;,$$

where the last equality can be seen by inserting *g*_*ab*_ = *x*_*a*_*x*_*b*_ + *y*_*a*_*y*_*b*_ (for all indices) and expanding. Taking one trace, i.e., contracting with *g*^*cd*^ gives $W_{ab}=\frac{2\alpha \beta }{3}\;g_{ab}$, and another trace gives $W=\frac{4\alpha \beta }{3}$, which proves the lemma. □

## 3 *R*_*abcd*_ as a Quadratic Form on $R^{3}$

Through the orthonormal basis for the space of symmetric two-tensors (in two dimensions) given by (2), the tensor *R*_*abcd*_ viewed as a quadratic form can be represented by a 3 × 3-matrix. Here, we will restrict ourselves to an orthonormal basis for *V*_(*ab*)_, namely the basis $\left\{e_{ab}^{\left(1\right)},e_{ab}^{\left(2\right)},e_{ab}^{\left(3\right)}\right\}$ from Sect. 2.1.3, defined in terms of the orthonormal basis [*ξ*^*a*^, *η*^*a*^} for *V*^*a*^. Thus, given *R*_*abcd*_, we associate the symmetric matrix *M_ij_*, where (the choice of an orthonormal basis justifies the mismatch of the indices *i, j*)

$$M_{ij}≐R_{\;\;cd}^{ab}e_{ab}^{\left(i\right)}(e^{\left(j\right)}{)}^{cd},\;\;1\leq i,\;j\leq 3.$$

It is instructive to see how the various derived tensors show up in *M*_*ij*_. In terms of the basis (2) it is natural to look at the various parts of *M*_*ij*_ as follows

(10) $$M_{ij}≐\left(\begin{array}{ccc} \times & \times & \times \\ \times & \times & \times \\ \times & \times & ✕ \end{array}\right)≐\left(\begin{array}{cc} A & \bar{v}\\ {\bar{v}}^{t} & a \end{array}\right).$$

This splitting is natural for reasons which will become apparent in the next sections. Note, however, that with this representation it is tempting to consider coordinate changes in $R^{3}$, which is not natural in this case. Rather, of interest is the change of basis in *V*^*a*^ and the related *induced* change of coordinates in the representation (10). See Sect. 3.2.

### 3.1 Representation of the Canonically Derived Parts of R_abcd_

It is helpful to see how the components of the various tensors *T*_*ab*_, *S*_*ab*_*, T*, *S*, ${\overset{{}_{\;{}_{\circ }}}{H}}_{abcd}$, ${\overset{{}_{\;{}_{\circ }}}{H}}_{ab}$, *H* and *W* show up as components of *M*_*ij*_. As for ${\overset{{}_{\;{}_{\circ }}}{H}}_{ab}$, e.g., ${\overset{{}_{{}_{\circ }}}{T}}_{ab}$ denotes the trace-free part of *T*_*ab*_. Immediate is *M*_33_:

(11) $$M_{33}≐R_{\;\;cd}^{ab}e_{ab}^{\left(3\right)}(e^{\left(3\right)}{)}^{cd}≐\frac{1}{2}R_{\;\;cd}^{ab}g_{ab}g^{cd}=\frac{1}{2}T_{cd}g^{cd}=\frac{1}{2}T.$$

Similarly, for *i* = 1, 2 we have

(12) $$M_{i3}≐\frac{1}{\sqrt{2}}R_{\;\;cd}^{ab}e_{ab}^{\left(i\right)}g^{cd}≐\frac{1}{\sqrt{2}}T^{ab}e_{ab}^{\left(i\right)}≐\frac{1}{\sqrt{2}}{\overset{{}_{{}_{\circ }}}{T}}^{ab}e_{ab}^{\left(i\right)},$$

where the last equality follows form the trace-freeness of $e_{ab}^{\left(1\right)}$ and $e_{ab}^{\left(2\right)}$. This means that the components of ${\overset{{}_{{}_{\circ }}}{T}}_{ab}$ (properly rescaled) goes into *M*_*ij*_ as the components of $\bar{v}$ (and ${\bar{v}}^{t}$) in (10). The same holds for ${\overset{{}_{\;{}_{\circ }}}{S}}_{ab}$ and ${\overset{{}_{\;{}_{\circ }}}{H}}_{ab}$, as ${\overset{{}_{\;{}_{\circ }}}{S}}_{ab}={\overset{{}_{{}_{\circ }}}{T}}_{ab}$ by Lemma 1, which then implies that also ${\overset{{}_{\;{}_{\circ }}}{H}}_{ab}={\overset{{}_{{}_{\circ }}}{T}}_{ab}={\overset{{}_{\;{}_{\circ }}}{S}}_{ab}$. This latter relation follows from the trace-free part of the relation $H_{ab}=\frac{1}{3}(T_{ab}+2S_{ab})$. Hence

(13) $$M_{ij}≐\left(\begin{array}{cc} A & \vec{\overset{{}_{{}_{\circ }}}{T}}\\ \vec{\overset{{}_{{}_{\circ }}}{T}}{\;}^{{}^{{}^{t}}} & \frac{1}{2}T \end{array}\right)≐\left(\begin{array}{cc} \frac{\sigma }{2}I+Å & \vec{\overset{{}_{{}_{\circ }}}{T}}\\ \vec{\overset{{}_{{}_{\circ }}}{T}}{\;}^{{}^{{}^{t}}} & \frac{1}{2}T \end{array}\right),$$

where $\vec{\overset{{}_{{}_{\circ }}}{T}}=\vec{\overset{{}_{\;{}_{\circ }}}{S}}=\vec{\overset{{}_{\;{}_{\circ }}}{H}}$ encodes the two degrees of freedom in ${\overset{{}_{{}_{\circ }}}{T}}_{ab}={\overset{{}_{\;{}_{\circ }}}{S}}_{ab}={\overset{{}_{\;{}_{\circ }}}{H}}_{ab}$. The matrix *A* is decomposed as $A=\frac{\sigma }{2}I+Å$ where *I* is the (2 × 2) identity matrix and *Å* is trace-free part of *A*. In particular, [*A*] = *σ*.

To investigate [*M*_*ij*_] = *M*_11_ + *M*_22_ + *M*_33_, i.e., the trace of *M*_*ij*_ we note that for a general symmetric matrix $R_{ij}≐\left(\begin{array}{cc} a & b\\ b & c \end{array}\right)$ we have $R_{ij}e_{ij}^{\left(1\right)}≐\frac{a-c}{\sqrt{2}}$, $R_{ij}e_{ij}^{\left(2\right)}≐\frac{2b}{\sqrt{2}}$, $R_{ij}e_{ij}^{\left(3\right)}≐\frac{a+c}{\sqrt{2}}$. When *M*_*ij*_ is constructed from *R*_*abcd*_ which is an outer product *R_ab_R_cd_* the trace is given by $M_{11}+M_{22}+M_{33}=(\frac{a-c}{\sqrt{2}}{)}^{2}+(\frac{2b}{\sqrt{2}}{)}^{2}+(\frac{a+c}{\sqrt{2}}{)}^{2}=a^{2}+2b^{2}+c^{2}$ and from (6) this is *S*. Together with linearity, this shows that [*M*] = *M*_11_ + *M*_22_ + *M*_33_ = *S* also when *R*_*abcd*_ is formed as in (1). Taking trace in (13), this gives

$$S=\sigma +\frac{1}{2}T,\;\;\text{i.e.},\;\;\sigma =S-\frac{1}{2}T\;.$$

In addition, the relations below Eq. (7) show that

{H=13(T+2S)W=23(T−S)}i.e.,{T=H+WS=H−12W}so thatσ=12H−W.

The two degres of freedom in *Å* corresponds to the two degrees of freedom in ${\overset{{}_{\;{}_{\circ }}}{H}}_{abcd}$.

### 3.2 The Behaviour of M_ij_ Under a Rotation of the Coordinate System in V^a^

The components of *M*_*ij*_ are expressed in terms of the orthonormal basis tensors given by (2), and these in turn are based on the ON basis $\left\{\hat{\xi },\hat{\eta }\right\}$ for *V*. Putting the basis vectors in a row matrix $\left(\hat{\xi }\;\hat{\eta }\right)$ and the coordinates in a column matrix $\left(\begin{array}{c} \xi \\ \eta \end{array}\right)$ so that a vector $u=\xi \hat{\xi }+\eta \hat{\eta }=\left(\hat{\xi }\;\hat{\eta }\right)\left(\begin{array}{c} \xi \\ \eta \end{array}\right)$, and considering only orthonormal frames, the relevant change of basis is given by a rotation matrix $Q(v)=Q_{v}=\left(\begin{array}{cc} \cos\;v & -\;\sin\;v\\ \sin\;v & \cos\;v \end{array}\right)$, i.e., we consider the change of basis

$$(\hat{\xi }\;\hat{\eta })\to \left(\hat{\tilde{\xi }}\;\hat{\tilde{\eta }}\right)=(\hat{\xi }\;\hat{\eta })\left(\begin{array}{cc} \cos\;v & -\;\sin\;v\\ \sin\;v & \cos\;v \end{array}\right)=(\hat{\xi }\;\hat{\eta })\;Q(v).$$

This means that for a vector $u=\left(\hat{\tilde{\xi }}\;\hat{\tilde{\eta }}\right)\left(\begin{array}{c} \tilde{\xi }\\ \tilde{\eta } \end{array}\right)=(\hat{\xi }\;\hat{\eta })\left(\begin{array}{c} \xi \\ \eta \end{array}\right)$, the coordinates transform as

$$\left(\begin{array}{c} \xi \\ \eta \end{array}\right)\to \left(\begin{array}{c} \tilde{\xi }\\ \tilde{\eta } \end{array}\right)=Q^{-1}(v)\left(\begin{array}{c} \xi \\ \eta \end{array}\right)=Q^{t}(v)\left(\begin{array}{c} \xi \\ \eta \end{array}\right)=Q(-v)\left(\begin{array}{c} \xi \\ \eta \end{array}\right).$$

For the components of the basis vectors $e_{ab}^{\left(1\right)}$, $e_{ab}^{\left(2\right)}$, $e_{ab}^{\left(3\right)}$ we find (omitting the factor $1\;∕\;\sqrt{2}$)

(14) $$\left(\begin{array}{cc} 1 & 0\\ 0 & -1 \end{array}\right)\to \left(\begin{array}{cc} \cos\;v & \sin\;v\\ -\;\sin\;v & \cos\;v \end{array}\right)\left(\begin{array}{cc} 1 & 0\\ 0 & -1 \end{array}\right)\left(\begin{array}{cc} \cos\;v & -\;\sin\;v\\ \sin\;v & \cos\;v \end{array}\right)=\left(\begin{array}{cc} \cos\;2v & -\;\sin\;2v\\ -\;\sin\;2v & -\;\cos\;2v \end{array}\right)\;\;\left(\begin{array}{cc} 0 & 1\\ 1 & 0 \end{array}\right)\to \left(\begin{array}{cc} \cos\;v & \sin\;v\\ -\;\sin\;v & \cos\;v \end{array}\right)\left(\begin{array}{cc} 0 & 1\\ 1 & 0 \end{array}\right)\left(\begin{array}{cc} \cos\;v & -\;\sin\;v\\ \sin\;v & \cos\;v \end{array}\right)=\left(\begin{array}{cc} \sin\;2v & \cos\;2v\\ \cos\;2v & -\;\sin\;2v \end{array}\right)\;\;\left(\begin{array}{cc} 1 & 0\\ 0 & 1 \end{array}\right)\to \left(\begin{array}{cc} \cos\;v & \sin\;v\\ -\;\sin\;v & \cos\;v \end{array}\right)\left(\begin{array}{cc} 1 & 0\\ 0 & 1 \end{array}\right)\left(\begin{array}{cc} \cos\;v & -\;\sin\;v\\ \sin\;v & \cos\;v \end{array}\right)=\left(\begin{array}{cc} 1 & 0\\ 0 & 1 \end{array}\right),$$

and this means that the components *M*_*ij*_ transform as

(15) $$M_{ij}≐\left(\begin{array}{cc} A & \bar{v}\\ {\bar{v}}^{t} & a \end{array}\right)\to {\tilde{M}}_{ij}≐\left(\begin{array}{cc} Q_{2v}^{t}A\;Q_{2v} & Q_{2v}^{t}\bar{v}\\ {\bar{v}}^{t}\;Q_{2v} & a \end{array}\right).$$

But this latter expression is just

$$\left(\begin{array}{cc} Q_{2v}^{t} & \bar{0}\\ {\bar{0}}^{t} & 1 \end{array}\right)\left(\begin{array}{cc} A & \bar{v}\\ {\bar{v}}^{t} & a \end{array}\right)\left(\begin{array}{cc} Q_{2v} & \bar{0}\\ {\bar{0}}^{t} & 1 \end{array}\right),$$

hence we have the following important remark/observation:

**Remark 1** Viewing the matrix *M*_*ij*_ as an ellipsoid in $R^{3}$, the effect of a rotation by an angle *v* in *V*^*a*^ corresponds to a rotation of the ellipsoid by an angle 2*v* around the *z*-axis in $R^{3}$ (where the *z*-axis corresponds to the ‘isotropic direction’ given by *g*_*ab*_).

## 4 The Equivalence Problem for *R*_*abcd*_

The equivalence problem for *R*_*abcd*_ can be formulated in different ways (for an account in three dimensions, we refer to [3]). Given two tensors *R*_*abcd*_ and ${\tilde{R}}_{abcd}$, both with the symmetries implied by (1), the question whether they are the same or not is straightforward as one can compare the components in any basis. However, *R*_*abcd*_ and ${\tilde{R}}_{abcd}$ could live in different (but isomorphic) vector spaces, e.g. two tangent spaces at different points, and the concept of equality becomes less clear. On the other hand, in terms of components *R*_*ijkl*_ and ${\tilde{R}}_{ijkl}$, one could ask whether there is a change of coordinates which takes one set of components into the other. If so, one can find a (invertible) matrix *P*^*i*^_*j*_ so that

$$R_{ijkl}={\tilde{R}}_{mnop}{P^{m}}_{i}{P^{n}}_{j}{P^{o}}_{k}{P^{p}}_{l},$$

and the tensors are then said to be equivalent. As already mentioned, it is convenient to restrict the coordinate systems to orthonormal coordinates. This means that two different coordinate systems differ only by their orientation, i.e., the change of coordinates are given by a rotation matrix *Q* ∈ SO(2). Under the ‘Cartesian convention’ that all indices are written as subscripts, *R*_*abcd*_ and ${\tilde{R}}_{abcd}$ are equivalent if there is a matrix *Q* ∈ SO(2) so that (their Cartesian components satisfy)

$$R_{ijkl}={\tilde{R}}_{mnop}Q_{mi}Q_{nj}Q_{ok}Q_{pl}.$$

### 4.1 Different Ways to Characterize the Equivalence of R_abcd_ and ${\tilde{R}}_{abcd}$

In this section, we will discuss three ways to determine whether two tensors *R*_*abcd*_ and ${\tilde{R}}_{abcd}$ are equivalent or not. In Sects. 4.1.1 and 4.1.2 we present two such methods briefly, while Sect. 4.1.3, which is more complete, contains the main result of this work.

As mentioned in Sect. 1.1, the results of Sects. 4.1.1 and 4.1.2, which may be used in their own rights, rely on particular choices of basis matrices for *V*_(*ab*)_. The formulation in Sect. 4.1.3 on the other hand, is expressed in the components of *R*_*abcd*_ (in any coordinate system) directly.

#### 4.1.1 Orientation of the Ellipsoid in $R^{3}$

A necessary condition for *R*_*abcd*_ and ${\tilde{R}}_{abcd}$ to be equivalent is that their corresponding 3 × 3-matrices *M*_*ij*_ and ${\tilde{M}}_{ij}$ have the same eigenvalues. On the other hand, this is not sufficient since the representation in $R^{3}$ should reflect the freedom in rotating the coordinate system in $V^{a}∼R^{2}$. With the coordinates adopted, this corresponds to a rotation of the associated ellipsoid around the *z*-axis in $R^{3}$ (see Remark 1 in Sect. 3.2). This is illustrated in Fig. 2 where three ellipsoids, all representing positive definite symmetric mappings having identical eigenvalues, are shown. The two first ellipsoids can be rotated into each other by a rotation around the *z*-axis. This implies that the corresponding tensors *R*_*abcd*_ and ${\tilde{R}}_{abcd}$ are equivalent. The third ellipsoid can also be rotated into the two others, but these rotations are around directions other than the *z*-axis, which means that this ellipsoid represents a different tensor.

In the generic case, with all eigenvalues different, it is easy to test whether two different ellipsoids can be transfered into each other through a rotation around the *z*-axis. This will be the case if the corresponding eigenvectors (of *M_ij_* and ${\tilde{M}}_{ij}$) have the same angle with the *z*-axis. Hence it is just a matter of checking the *z*-components of the three normalized eigenvectors and see if they are equal up to sign.

#### 4.1.2 Components in a Canonical Coordinate System

In a sense, this is the most straightforward method. In a coordinate system which respects $e_{ab}^{\left(3\right)}$ as the *z*-axis in $V_{\left(ab\right)}∼R^{3}$, two tensors *R*_*abcd*_ and ${\tilde{R}}_{abcd}$ are equivalent if there is a rotation matrix (in two dimensions) *Q* such that

(16) $$\left(\begin{array}{cc} A & \vec{\overset{{}_{{}_{\circ }}}{T}}\\ \vec{\overset{{}_{{}_{\circ }}}{T}}{\;}^{{}^{{}^{t}}} & \frac{1}{2}T \end{array}\right)=\left(\begin{array}{cc} Q^{t}\tilde{A}Q & Q^{t}\vec{\overset{{}_{\;{}_{\circ }}}{\tilde{T}}}\\ \vec{\overset{{}_{\;{}_{\circ }}}{\tilde{T}}}{\;}^{{}^{{}^{t}}}Q & \frac{1}{2}\tilde{T} \end{array}\right).$$

Hence, equivalence can be easily tested by first checking that $T=\tilde{T}$ and that $\left\|\vec{\overset{{}_{{}_{\circ }}}{T}}\|=\|\vec{\overset{{}_{{}_{\circ }}}{\tilde{T}}}\right\|$. If this is the case, (and if $\|\vec{\overset{{}_{{}_{\circ }}}{T}}\|>0$) one determines the rotation matrix *Q* which gives $\vec{\overset{{}_{{}_{\circ }}}{T}}=Q^{t}\vec{\overset{{}_{{}_{\circ }}}{\tilde{T}}}$, and equivalence is then determined by if $A=Q^{t}\tilde{A}Q$ or not. If $\|\vec{\overset{{}_{{}_{\circ }}}{T}}\|=\|\vec{\overset{{}_{{}_{\circ }}}{\tilde{T}}}\|=0$, the equivalence of *A* and $\tilde{A}$ can be determined directly, i.e., by checking whether $\left[A]=[\tilde{A}\right]$ and $\left[A^{2}]=[{\tilde{A}}^{2}\right]$ or not.

#### 4.1.3 Equivalence Through (algebraic) Invariants of *R*_*abcd*_

If a solution is found, this is perhaps the most satisfactory way to establish equivalence, in particular if the invariants are constructed by simple algebraic operations only. (For instance, to a symmetric 3 × 3-matrix *A* one can take the three eigenvalues as invariants or else for instance the traces of *A*, *A*^2^ and *A*^3^. The former set requires some calculations, but the latter is immediate.)

Examples of invariants are *T* = *R*_*abcd*_*g*^*ab*^*g*^*cd*^, *S* = *R*_*abcd*_*g*^*ac*^*g*^*bd*^ and the invariants *H* = *H*_*ab*_*g*^*ab*^, *W* = *W*_*ab*_*g*^*ab*^. To produce the invariants, we use the tensor *R*_*abcd*_ and the metric *g*_*ab*_. However, if we regard $V^{a}∼R^{2}$ as oriented, so that the orthonormal basis $\left\{\hat{\xi },\hat{\eta }\right\}$ for *V*^*a*^ also is oriented, then invariants can also be formed in another way. Namely, since the space of symmetric 2 × 2 matrices is 3-dimensional, and since the metric *g_ab_* singles out a 1-dimensional subspace, it also determines a 2-dimensional subspace *L*; all elements orthogonal to *g*_*ab*_. This subspace is the set of all symmetric 2 × 2 matrices which are also trace-free. *L* can be given an orientation through an area form, which in turn inherits the orientation from *V*^*a*^.

In general, with right-handed Cartesian coordinates *x*^1^, *x*^2^, the area form *ϵ* is given by *ϵ* = *dx*^1^ ∧ *dx*^2^ where (*ω* ∧ *μ*)_*ab*_ = *ω_a_μ_b_* − *ω_b_μ_a_*. With the orthonormal basis $\left\{\hat{\xi },\hat{\eta }\right\}$ (for *V*^*a*^ ) also right handed, we define, cf. (2),

(17) $$e_{ab}^{\left(1\right)}=\frac{1}{\sqrt{2}}({\hat{\xi }}_{a}{\hat{\xi }}_{b}-{\hat{\eta }}_{a}{\hat{\eta }}_{b}),\;\;e_{ab}^{\left(2\right)}=\frac{1}{\sqrt{2}}({\hat{\xi }}_{a}{\hat{\eta }}_{b}+{\hat{\eta }}_{a}{\hat{\xi }}_{b}).$$

The area form on *L* is then *ϵ* ~ *e*^(1)^ ∧ *e*^(2)^, or

(18) $$\epsilon ∼E_{abcd}=e_{ab}^{\left(1\right)}e_{cd}^{\left(2\right)}-e_{ab}^{\left(2\right)}e_{cd}^{\left(1\right)}.$$

It is not hard to see that this definition is independent of the orientation of $\left\{\hat{\xi },\hat{\eta }\right\}$. We observe that $2E_{abcd}=({\hat{\xi }}_{a}{\hat{\xi }}_{b}-{\hat{\eta }}_{a}{\hat{\eta }}_{b})({\hat{\xi }}_{c}{\hat{\eta }}_{d}+{\hat{\eta }}_{c}{\hat{\xi }}_{d})-({\hat{\xi }}_{a}{\hat{\eta }}_{b}+{\hat{\eta }}_{a}{\hat{\xi }}_{b})({\hat{\xi }}_{c}{\hat{\xi }}_{d}-{\hat{\eta }}_{c}{\hat{\eta }}_{d})$. By replacing $\hat{\xi }$ by $\hat{\omega }=\cos\;v\;\hat{\xi }+\sin\;v\;\hat{\eta }$ and $\hat{\eta }$ by $\hat{\mu }=-\;\sin\;v\;\hat{\xi }+\cos\;v\;\hat{\eta }$, i.e., a rotated orthonormal basis, it is straightforward to check that

(19) $$\left({\hat{\omega }}_{a}{\hat{\omega }}_{b}-{\hat{\mu }}_{a}{\hat{\mu }}_{b})({\hat{\omega }}_{c}{\hat{\mu }}_{d}+{\hat{\mu }}_{c}{\hat{\omega }}_{d})-({\hat{\omega }}_{a}{\hat{\mu }}_{b}+{\hat{\mu }}_{a}{\hat{\omega }}_{b})({\hat{\omega }}_{c}{\hat{\omega }}_{d}-{\hat{\mu }}_{c}{\hat{\mu }}_{d})\;=({\hat{\xi }}_{a}{\hat{\xi }}_{b}-{\hat{\eta }}_{a}{\hat{\eta }}_{b})({\hat{\xi }}_{c}{\hat{\eta }}_{d}+{\hat{\eta }}_{c}{\hat{\xi }}_{d})-({\hat{\xi }}_{a}{\hat{\eta }}_{b}+{\hat{\eta }}_{a}{\hat{\xi }}_{b})({\hat{\xi }}_{c}{\hat{\xi }}_{d}-{\hat{\eta }}_{c}{\hat{\eta }}_{d}\right)$$

so that *E*_*abcd*_ is well defined. We recollect that area form *E*_*abcd*_ is defined, through the induced metric, on the plane *L* (which in turn is also defined through the metric *g*_*ab*_) and the orientation on *V*^*a*^. Hence *E*_*abcd*_ can be used when forming invariants.

We will now state the result of this work, namely the existence of six invariants which can be used to investigate equivalence of two tensors *R*_*abcd*_ and ${\tilde{R}}_{abcd}$. We start by defining

(20) $$S=R_{abcd}g^{ac}g^{bd}\;T=R_{abcd}g^{ab}g^{cd}\;J_{0}=R_{abcd}R^{abcd}\;J_{1}=T^{ab}T_{ab}\;J_{2}=R_{abcd}T^{ab}T^{cd}\;J_{3}=T^{ab}R_{abcd}E^{cdef}T_{ef}.$$

where *E*_*abcd*_ is defined by (17) and (18). Similarly, we define $\tilde{S}$, $\tilde{T}$, ${\tilde{J}}_{0}$, ${\tilde{J}}_{1}$, ${\tilde{J}}_{2}$ and ${\tilde{J}}_{3}$ as the corresponding invariants formed from ${\tilde{R}}_{abcd}$. We make the remark that for most of these invariants, their immediate interpretations still remain to be found. Rather, their value lie in the fact that they form a set which can be used to establish the equivalence in Theorem 1 below. On the other hand, some interpretations are possible. In particular, the quotient *T/S* (see Definition 1) lies in the interval [1, 2] and has the meaning given by Lemma 2.

**Theorem 1** *Suppose that* $R_{abcd}=\sum_{i=1}^{n}R_{ab}^{\left(i\right)}R_{cd}^{\left(i\right)}$, *with* $R_{ab}^{\left(i\right)}\geq 0$ *and that R_ijkl_ are the components of R_abcd_ in some basis. Suppose also that* ${\tilde{R}}_{abcd}=\sum_{i=1}^{\tilde{n}}{\tilde{R}}_{ab}^{\left(i\right)}{\tilde{R}}_{cd}^{\left(i\right)}$, *with* ${\tilde{R}}_{ab}^{\left(i\right)}\geq 0$ *and that* ${\tilde{R}}_{ijkl}$ *are the components of* ${\tilde{R}}_{abcd}$ *in some, possibly unrelated, basis. If (and only if)* $S=\tilde{S}$, $T=\tilde{T}$, $J_{0}={\tilde{J}}_{0}$, $J_{1}={\tilde{J}}_{1}$, $J_{2}={\tilde{J}}_{2}$, $J_{3}={\tilde{J}}_{3}$, *then there is a transformation matrix P^i^_j_ such that*

$$R_{ijkl}={\tilde{R}}_{mnop}{P^{m}}_{i}{P^{n}}_{j}{P^{o}}_{k}{P^{p}}_{l}.$$

***Proof*** Since the invariants are defined without reference to any basis, it is sufficient to consider the components expressed in an orthonormal frame, and in that case we must prove the existence of a rotation matrix *Q* ∈ SO(2) so that

$$R_{ijkl}={\tilde{R}}_{mnop}Q_{mi}Q_{nj}Q_{ok}Q_{pl}.$$

Since

(21) $$R_{abcd}=M_{ij}e_{ab}^{\left(i\right)}e_{cd}^{\left(j\right)},$$

we can consider the invariants formed from the components of

(22) $$M_{ij}=\left(\begin{array}{cc} A & \bar{u}\\ {\bar{u}}^{t} & c \end{array}\right)\;\text{and}\;{\tilde{M}}_{ij}=\left(\begin{array}{cc} \tilde{A} & \tilde{\bar{u}}\\ {\tilde{\bar{u}}}^{t} & \tilde{c} \end{array}\right)$$

and we must demonstrate the existence of a rotation matrix *Q* = *Q*_2*v*_ such that

(23) $$\tilde{A}=Q_{2v}^{t}AQ_{2v},\;\;\tilde{\bar{u}}=Q_{2v}^{t}\bar{u},\;\;\tilde{c}=c\;.$$

We make the ansatz

(24) $$M_{ij}=\left(\begin{array}{cc} \begin{array}{c} \frac{\sigma }{2}+a\\ b \end{array}\begin{array}{c} b\\ \frac{\sigma }{2}-a \end{array} & \begin{array}{c} x\\ y \end{array}\\ x\;y & c \end{array}\right),\;\;{\tilde{M}}_{ij}=\left(\begin{array}{cc} \begin{array}{c} \frac{\tilde{\sigma }}{2}+\tilde{a}\\ \tilde{b} \end{array}\begin{array}{c} \tilde{b}\\ \frac{\tilde{\sigma }}{2}-\tilde{a} \end{array} & \begin{array}{c} \tilde{x}\\ \tilde{y} \end{array}\\ \tilde{x}\;\tilde{y} & \tilde{c} \end{array}\right).$$

Through (21) it is straightforward to see that

$$S=\sigma +c,\;\;T=2c,\;\;J_{0}=2(a^{2}+b^{2})+c^{2}+\sigma^{2}∕2+2(x^{2}+y^{2}),\;J_{1}=2(c^{2}+x^{2}+y^{2})$$

so if $S=\tilde{S}$, $T=\tilde{T}$, $J_{0}={\tilde{J}}_{0}$, $J_{1}={\tilde{J}}_{1}$, it follows that $\sigma =\tilde{\sigma }$, $c=\tilde{c}$, $a^{2}+b^{2}={\tilde{a}}^{2}+{\tilde{b}}^{2}$ and $x^{2}+y^{2}={\tilde{x}}^{2}+{\tilde{y}}^{2}$. Since the isotropic part of *A*, i.e., $\frac{\sigma }{2}I$ is unaffected by a rotation of the coordinate system, we consider the traceless parts $Å=\left(\begin{array}{cc} a & b\\ b & -a \end{array}\right)$, $\overset{\;{}_{\circ }}{\tilde{A}}=\left(\begin{array}{cc} \tilde{a} & \tilde{b}\\ \tilde{b} & -\tilde{a} \end{array}\right)$, and the task is to assert a rotation matrix *Q* such that

$$\left(\begin{array}{cc} a & b\\ b & -a \end{array}\right)=Q^{t}\left(\begin{array}{cc} \tilde{a} & \tilde{b}\\ \tilde{b} & -\tilde{a} \end{array}\right)Q,\;\;\left(\begin{array}{c} x\\ y \end{array}\right)=Q^{t}\left(\begin{array}{c} \tilde{x}\\ \tilde{y} \end{array}\right),$$

if also $J_{2}={\tilde{J}}_{2}$, $J_{3}={\tilde{J}}_{3}$. Again it is straightforward to calculate the remaining invariants, and we find

$$J_{2}=4bxy+2a(x^{2}-y^{2})+2c^{3}+(4c+\sigma )(x^{2}+y^{2})\;J_{3}=4axy-2b(x^{2}-y^{2})\;.$$

and similarly for ${\tilde{J}}_{2}$, ${\tilde{J}}_{3}$. Hence, (since $\sigma =\tilde{\sigma }$, $c=\tilde{c}$)

(25) $$\;a^{2}+b^{2}={\tilde{a}}^{2}+{\tilde{b}}^{2}\;\;x^{2}+y^{2}={\tilde{x}}^{2}+{\tilde{y}}^{2}\;2bxy+a(x^{2}-y^{2})=2\tilde{b}\tilde{x}\tilde{y}+\tilde{a}({\tilde{x}}^{2}-{\tilde{y}}^{2})\;2axy-b(x^{2}-y^{2})=2\tilde{a}\tilde{x}\tilde{y}-\tilde{b}({\tilde{x}}^{2}-{\tilde{y}}^{2})\;.$$

Suppose first that *x*^2^ + *y*^2^ > 0. The equality $x^{2}+y^{2}={\tilde{x}}^{2}+{\tilde{y}}^{2}$ then guarantees the existence of the rotation matrix *Q* which is determined via the relation $\left(\begin{array}{c} x\\ y \end{array}\right)=Q^{t}\left(\begin{array}{c} \tilde{x}\\ \tilde{y} \end{array}\right)$. This can also be expressed as $Q_{1}^{t}\left(\begin{array}{c} x\\ y \end{array}\right)=Q_{2}^{t}\left(\begin{array}{c} \tilde{x}\\ \tilde{y} \end{array}\right)$ for some rotation matrices *Q*_1_, *Q*_2_, where $Q=Q_{2}Q_{1}^{t}$. We now choose the rotation matrix *Q*_1_ so that in the untilded coordinates, *y* = 0. Similarly we choose *Q*_2_ so that for the tilded coordinates, we get a frame where $\tilde{y}=0$. The equalities between the invariants in (25) then become

$$a^{2}+b^{2}={\tilde{a}}^{2}+{\tilde{b}}^{2}\;\;x^{2}={\tilde{x}}^{2}\;\;ax^{2}=\tilde{a}{\tilde{x}}^{2}\;\;-bx^{2}=-\tilde{b}{\tilde{x}}^{2}\;,$$

so that $a=\tilde{a}$, $b=\tilde{b}$. This proves the theorem when *x*^2^ + *y*^2^ > 0. When $x^{2}+y^{2}={\tilde{x}}^{2}+{\tilde{y}}^{2}=0$, i.e., $x=y=\tilde{x}=\tilde{y}=0$, the remaining equality $a^{2}+b^{2}={\tilde{a}}^{2}+{\tilde{b}}^{2}$ is sufficient since we can again choose frames in which $b=\tilde{b}=0$ and $a>0,\;\tilde{a}>0$. It then follows that $a=\tilde{a}$. □

## 5. Discussion

In this work, we started with a family of symmetric positive (semi-)definite tensors in two dimensions and considered its variance. This lead us to a fourth order tensor *R_abcd_* with the same symmetries as the elasticity tensor in continuum mechanics. After listing a number of possible issues to address, we focused on the equivalence problem. Namely, given the components of two such tensors *R*_*abcd*_ and ${\tilde{R}}_{abcd}$, how can one determine if they represent the same tensor (but in different coordinate systems) or not? In Sect. 4, we saw that this could be investigated in different ways. The result of Theorem 1 is most satisfactory in the sense that it is expressible in terms of the components of the fourth order tensors directly.

There are two natural extensions and/or ways to continue this work. The first is to apply the result to realistic families of e.g., diffusion tensors in two dimensions. The objective is then, apart from establishing possible equivalences, to investigate the geometric meaning of the invariants. The other natural continuation is to investigate the corresponding problem in three dimensions. The degrees of freedom of *R*_*abcd*_ will then increase from 6 to 21, leaving us with a substantially harder, but also perhaps more interesting, problem.

## Footnotes

1 For the notation of tensors used here, see Sect. 2.1.
